# Development of a tailored intervention targeting sedentary behavior and physical activity in people with stroke and diabetes: A qualitative study using a co-creation framework

**DOI:** 10.3389/fresc.2023.1114537

**Published:** 2023-02-13

**Authors:** Stefan Sjørslev Bodilsen, Mette Aadahl, Troels Wienecke, Trine Hørmann Thomsen

**Affiliations:** ^1^ Department of Neurology, Zealand University Hospital, Roskilde, Denmark; ^2^ Department of Physiotherapy and Occupational Therapy, Zealand University Hospital, Køge-Roskilde, Denmark; ^3^ Faculty of Health and Medical Science, University of Copenhagen, Copenhagen, Denmark; ^4^ Department of Clinical Medicine, University of Copenhagen, Copenhagen, Denmark; ^5^ Centre for Clinical Research and Prevention, Bispebjerg and Frederiksberg Hospital, Frederiksberg, Denmark; ^6^ Movement Disorder Clinic, Department of Brain - and Nerve Injuries, Rigshospitalet, Glostrup, Denmark; ^7^ The Parkinson's Association, The House of Disabled People's Organizations, Copenhagen, Denmark

**Keywords:** inactivity, rehabilitation, interview, activities of daily living, stroke, type 2 diabetes melitus (T2DM)

## Abstract

**Purpose:**

Type 2 diabetes and sedentary behavior pose serious health risks in stroke survivors. Using a co-creation framework, this study aimed to develop an intervention in collaboration with stroke survivors with type 2 diabetes, relatives, and cross-sectoral health care professionals to reduce sedentary behavior and increase physical activity.

**Materials and methods:**

This qualitative explorative study used a co-creation framework consisting of a workshop and focus group interviews with stroke survivors with type 2 diabetes (*n* = 3), relative (*n* = 1), and health care professionals (*n* = 10) to develop the intervention. A content analysis was used to analyze data.

**Results:**

The developed “Everyday Life is Rehabilitation” (ELiR) intervention consisted of a tailored 12-week home-based behavior change intervention with two consultations of action planning, goal setting, motivational interviewing, and fatigue management including education on sedentary behavior, physical activity, and fatigue. The intervention has a minimalistic setup using a double-page paper “Everyday Life is Rehabilitation” (ELiR) instrument making it implementable and tangible.

**Conclusions:**

In this study, a theoretical framework was used to develop a tailored 12-week home-based behavior change intervention. Strategies to reduce sedentary behavior and increase physical activity through activities of daily living along with fatigue management in stroke survivors with type 2 diabetes were identified.

## Introduction

Stroke and type 2 diabetes mellitus (T2DM) are both common diseases and among the top ten causes of disability worldwide (1) along with being some of the most costly diseases with expenses expected to increase (2–4). Stroke survivors with T2DM are at high risk of poor health and mortality compared to individuals living with only one of these diagnoses (5). T2DM poses a four times higher risk of stroke (6) and is an independent risk factor for stroke recurrence (5). Living with several health issues forces individuals to manage multiple negative consequences of their morbidities daily, such as impaired physical function and coordination of numerous interactions with the healthcare system. In addition, up to half of stroke survivors and individuals with T2DM experience fatigue (7, 8) and/or depression (9, 10). Added up these factors make this group particularly vulnerable, prone to sedentary behavior (SB) (11, 12), poor ability to perform activities of daily living (ADL) (13), and low quality of life (QoL) (13).

SB is associated with cardiovascular disease, T2DM, and premature death (14). Stroke survivors and individuals with T2DM spend more time with SB, are shown to have lower physical activity (PA) levels, and do not meet general PA guidelines compared to healthy peers (11, 12, 15, 16). In addition, stroke survivors with T2DM are more sedentary than stroke survivors without T2DM (17). PA is essential for preventing disability, improving physical function following stroke (18), and reducing mortality and morbidity in individuals with T2DM (19). WHO has recently emphasized the health benefits of PA and limiting SB for individuals living with disabilities (20).

Due to the health benefits, numerous interventions with different methods and contradicting results focus on reducing SB and increasing PA among stroke survivors or individuals with T2DM patients (21–31). In stroke survivors; no effect of light PA on insulin was reported (32), however low amounts of PA (33) and prolonged periods of SB >90 min were both found to increase HbA1c levels (17). These results provide an incentive to break up prolonged periods of SB and increase PA, however, this is not easy due to the complexity of the factors influencing SB and PA levels in stroke survivors and individuals with T2DM (34–38). Thus, it is important to explore which components should be included in multicomponent and tailored interventions (24, 39).

In recent years co-creation as a method, has become acknowledged for developing interventions when the development process is supported by behavior change theories (21, 40). One such theory, the Social Cognitive Theory by Albert Bandura evolves around aspects of the behavior itself along with personal and environmental factors, hereunder action planning and motivation (41). This theory has previously been used in co-creation processes (22, 26, 27) but co-creation frameworks have not been applied for a population of stroke survivors with T2DM (28). However, co-creation may be a feasible way to obtain a better understanding of SB and PA behaviors in this population as T2DM, SB and low levels of PA increase the risk of stroke (5, 6, 14), poor post-stroke recovery (13, 42), morbidity, and mortality (18, 19). Therefore, interventions that aim for beneficial effects of PA in stroke survivors with T2DM are desirable (43).

Using a co-creation framework, this study aimed to develop an intervention in collaboration with stroke survivors with T2DM, relatives, and cross-sectoral health care professionals (HCP) to reduce SB and increase PA.

## Methods

### Design

The co-creation of the intervention was based on the Social Cognitive Theory (41) and followed the framework by Leask et al. (44). Five principles from the systematic approach PRODUCES were included in this framework: (1) Framing the aim of the study; (2) Sampling; (3) Manifesting ownership; (4) Defining the procedure; and (5) Evaluating (the co-creation process) were utilized for this qualitative explorative study throughout a workshop and three focus group interviews. The mix of a workshop and focus group interviews contributes to a diverse understanding and allow participants to discuss and reflect on each other's experiences stimulating group interactions and dynamics (45). The role of the researchers and confidentiality within the group were clarified before beginning the workshop and focus group interviews.

This study was conducted at Neurovascular Center at Zealand University Hospital, Roskilde, Denmark (NC) between 07/02/2022–02/03/2022. This study followed the Consolidated Criteria for Reporting Qualitative Research (COREQ) checklist for reporting qualitative research (46) and the GUIDance for the reporting of intervention Development (GUIDED) (47).

### Participants

Eligible participants were recruited consecutively face to face from the NC. The inclusion criteria for stroke survivors with T2DM were ischemic stroke or intracerebral hemorrhage, diagnosed with T2DM by a specialist prior to their admission to NC, modified Rankin score (mRS) (48) 1–3 at discharge, discharged with a rehabilitation plan within 1–2 hospitalization days, able to ambulate independently, speak and understand Danish, able to give informed consent and motivated to contribute in a workshop and focus group interviews. Exclusion criteria were type 1 diabetes mellitus, dysphasia or cognitive impairments severe enough to preclude informed consent, medically unstable, considered too physically unstable by the clinical team to participate, or discharged to inpatient rehabilitation or a nursing home. The stroke survivors with T2DM were invited before discharge to the workshop and focus group interviews, which took place two to four weeks after discharge.

Relatives were recruited as they visited and/or picked up their relatives and included if they were related to an individual with the above-mentioned criteria and were able to speak and understand Danish. Included relatives and patients were not to be related.

Author SS engaged with management at NC and municipal rehabilitation centers and obtained permission to approach HCPs to request participation in the workshop and focus group interviews. Two occupational therapists (OTs), two physiotherapists (PTs), two nurses, and two stroke care coordinators working at stroke rehabilitation and linked community services were purposely invited. HCPs were included if they were working in stroke rehabilitation at a hospital or a municipal rehabilitation center, with more than three years of experience in stroke rehabilitation, and were able to speak and understand Danish.

### Workshop

The workshop took place in an auditorium with participants seated in a U shape facing a presentation screen at NC. Following the framework (44) the workshop started with describing the purpose, framing the session, and facilitating ownership of the co-creation process by underlining equal status and participation, emphasizing their responsibilities and encouraging openness and control of the process. Subsequently, prepared in a written script, the workshop consisted of open questions and exercises to explore the knowledge and perspectives on SB and PA. Later, picture presentations and scenarios were used to clarify perspectives, feelings, and opinions on lifestyle and rehabilitation. The picture presentation consisted of images of middle-aged people which the research team found reflected the body, PA, quality of life, health, diet, and thoughts on the future. The scenarios were concrete situations from everyday life e.g., on how the participants would break up prolonged SB or implement more movement in their ADL. After a break the participants' overall perspectives were represented and the generalization of results, user-friendliness, and feasibility of the future intervention were discussed and optimized from the participants' perspectives. All discussions were taken in plenary. Author SS functioned as interviewer and facilitated the workshop while co-author TT functioned as a mediator/facilitator and took field notes on general observations, content, and elements for further elaboration in the focus group interviews. The workshop was not audio recorded.

### Focus group interviews

For the focus group interviews, a meeting room was used with participants seated at a square table at NC. Participants were divided into three groups, one with stroke survivors with T2DM and relatives and two groups with HCPs. This was done as the stroke survivors with T2DM might feel less comfortable stating their opinions about their admission when HCP were present. Each group participated in one focus group interview. The focus group interviews were semi-structured, using the funnel model starting with broad questions before more specific questions (49), and focused on getting the participants to share and discuss opposite opinions and perspectives. Social dynamics and interactions between the participants were encouraged to create an informal atmosphere and get the participants to contribute actively and express as many different opinions and perspectives as possible (45).

All focus group interviews followed the interview guide (Supplementary file, S1) based on content and field notes from the workshop and previous literature (36, 37) with SS as interviewer and TT as co-interviewer. The guide provided the main structure, however, if relevant topics arose, the participants were encouraged to discuss and elaborate on them. The interviews were audio-recorded and TT took field notes on the atmosphere, interactions, reactions, and reflections. The stroke survivors with T2DM and relatives were asked about their daily living, views on SB, and, motivational factors for PA, and barriers to changing their lifestyle. HCPs were asked about their view on current rehabilitation, organizational factors, areas for improvement, lifestyle changes, and motivators for PA.

### Ethics

Ethical approval was obtained from Region Zealand Ethics Committee on 13/12/2021 (SJ950, EMN-2021-08261). This study complied with the Declaration of Helsinki and the General Data Protection Regulation (GDPR). All participants gave written informed consent and had no prior relation to the researchers or knowledge of this study.

### Analysis

**Figure 1:**
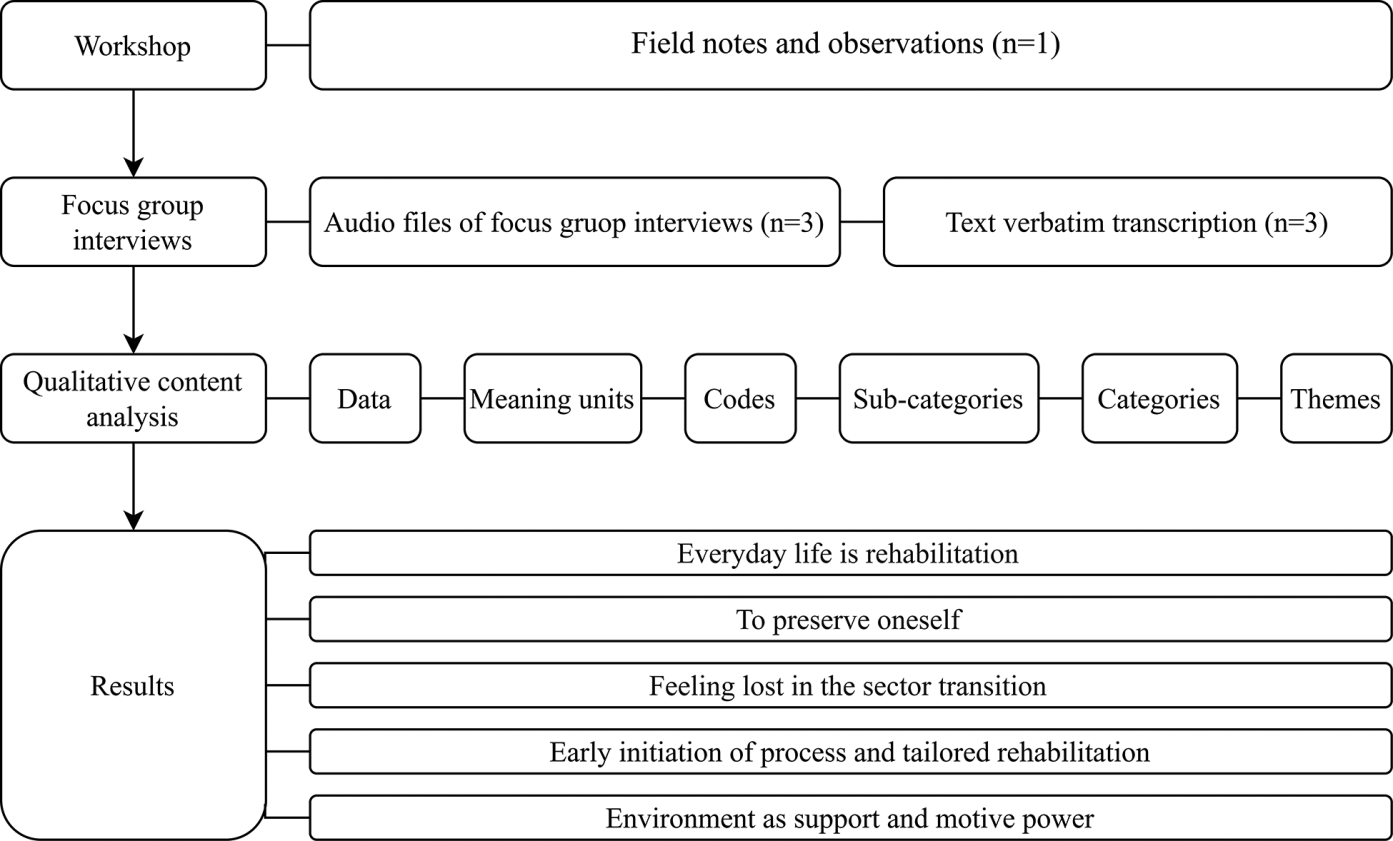
Methodology flowchart.

The focus group interviews were transcribed verbatim and pseudo-anonymized transcripts were analyzed using the content analysis method by Graneheim and Lundmann (50) alongside field notes. Data were inductively analyzed parallel by SS and TT in a triangulation process. Firstly, by familiarizing themselves with the data from the focus group interviews as a whole. Then separately focusing on manifest content using the complete focus group interview as a unit of analysis and afterwards comparing and agreeing upon the content. Abstracting meaning units into codes where first done separately then compared and agreed upon before continuing doing the same with sub-categories, categories, main categories, and lastly themes (Figure 1). Subsequently, SS and TT met to review consistency of abstraction levels, discuss categories, and condense these into themes for all focus group interviews. As no new coding items emerged when re-reading the meaning units, the analysis process continued with extraction of sub-categories, categories and themes. The research team translated the main themes from Danish into English. For transparency, Figure 1 provides an overview of the methodology and analysis process.

### Enhancing rigor

To ensure credibility and provide broad insights, a workshop and focus group interviews with three different participant groups were used. The researcher team was experienced in the field of stroke rehabilitation and/or in conducting qualitative research. The research team consists of SS; male physiotherapist and Ph.D. student with 5 years of experience in stroke rehabilitation, MA; female physiotherapist and clinical professor with more than 20 years of experience in SB, PA, and behavior change, TW; male MD and clinical associate professor with 14 years of experience in stroke and TT; female nurse and post.doc. with 19 years of clinical experience in neurology and 5 years of experience within mixed methods and conduct of everyday life. Collaboratively, before conducting this study, all activities, organization, and analysis were discussed addressing “pre-understanding”. SS and TT conducted the workshop and focus group interviews and critically reflected on data collection and validation of the findings during analysis. To enhance transferability a predefined description of the context and aim of the research and methodological considerations with notes on interpretations and decisions during the analysis were followed (51).

The analysis process was chosen in order to ensure transparency and to strengthen the credibility of the process (Figure 1 and Supplementary file, S2). Dependability was sought through describing methods as well as analytic strategies. To ensure confirmability, authors SB and TT repeatedly reread and reheard the interview material to stay close to the participants’ statements. As a part of this process, triangulation was intensively performed to challenge any pre-assumptions and misinterpretations to ensure trustworthiness (52).

## Findings

Three male stroke survivors with T2DM, one female relative, and 5 HCPs participated in the workshop. The same participants and 5 additional HCPs participated in three separate focus group interviews. One PT and one OT working as a stroke care coordinator were recruited from municipal rehabilitation section and one nurse from the Danish Stroke Association, an organization supporting individuals in life after stroke. The remaining stroke survivors with T2DM, relative, and HCP were recruited from NC. See Table 1 for characteristics of the stroke survivors with T2DM and HCP. The one relative participating in this study was a 76-year-old female, retired nurse, living with a male with an ischemic stroke (mRS of 1 with mild paralyses of his right arm).

**Table 1:** Stroke survivors with type 2 diabetes mellitus and health care professional's characteristics.

Characteristics, Stroke survivors with T2DM	Participants (*n* = 3)
Ischemic stroke, *n*	2
Intracerebral hemorrhage, *n*	1
Age in years, mean	77
Male, *n*	3
Affected right side, *n*	3
Used a walking aid, *n*	1
Cohabitants, *n*	2
Working status retired, *n*	3
Level of education above high school, *n*	1
Characteristics, health care professionals	Participants (*n* = 10)
Age in years, mean	39.9
Female, *n*	8
Male, *n*	2
Physiotherapist, *n*	4
Occupational therapist, *n*	3
Nurse, *n*	3
Level of education above bachelor's degree, *n*	1
Years of experience in stroke rehabilitation, mean	11.1

Thirteen stroke survivors with T2DM were eligible. Hereof nine were invited to participate, the remaining four were not invited due to other related examinations. Four stroke survivors with T2DM declined to attend and two dropped out, all describing it as unmanageable and overwhelming in their current situation, for example due to duration of transport to the hospital, time point, and duration of the workshop and focus group interviews. Three relatives were invited to participate in this study, as they were present at the ward. One declined due to work and one dropped out due to the date of the workshop and focus group interviews. Forty-four HCP were assessed for eligibility; thirteen did not meet inclusion criteria. Management at NC and municipal rehabilitation centers asked fifteen random HCPs if they would be interested in participating after which SS informed and invited them. Of the fifteen HCP's three declined to participate since the workshop and focus group interviews took place during their leisure time. Two dropped out due to COVID-19. All participants were recruited from December 2021 to February 2022.

The workshop lasted two hours and each of the three focus group interviews lasted approximately one hour. The full interview guide was used in all interviews. However, some items were discussed passionately by the stroke survivors with T2DM, including driving ban after stroke, discharge, sector transition, and the information procedure in the healthcare system, even though these items were not intently emphasized in the interview guide. Time for these discussions was allowed as they served as ice-breaking items/moments and led to new insights.

The concurrent analysis collectively for all interviews resulted in five overarching themes (1) *Everyday life is rehabilitation*, (2) *To preserve oneself*, (3) *Feeling lost in the sector transition*, (4) *Early initiation of process and tailored rehabilitation*, and (5) *Environment as support and motive power*. Each theme is presented with quotation examples below and in Supplementary file S2 showing steps of the analysis process and abstraction level from meaning units to themes.

The theme *Everyday life is rehabilitation* emerged from participants describing that the best way to ensure PA was to implement it into activities of everyday life. Overall, participants did not want to do more than they already do. Further participants described generic self-managed home-based exercises as overwhelming due to fatigue and lack of motivation and that PA should be rephrased to movement.

*Training of the hand, that's good enough, but fitness or something like that, no no it isn't me. What you see people do on television, what are they doing, bending and stretching and I don’t know what. That's not me. **Male patient, 75 years***

*This autumn, I will clear one side of my garden together with the neighbor. My board fence is broken, so now I have to dig down new posts … that's training. **Male patient 74 years***

*We also have fatigue among many of our citizens which play a part and which do that they can’t manage it all … so that's where they prioritize, and physical activity is very far down on the priority list. **Female municipal OT, 48 years***

*To preserve oneself* reflects a major motivator for the participants to engage in PA. Participants wanted to preserve their appearance both at home and in public and be able to perform their ADL independently. These factors seem to be closely linked to the feeling of being the same person as before the stroke and T2DM diagnosis. Basically, they wanted to keep living the life they knew and valued. However, fatigue was described as a barrier to achieving this.

*I don’t think I need to change anything… but having others to help me put on socks, that's very annoying at this point. **Male patient, 82 years***

*I think it means the most that you can decide for yourself, what you want to do and that you don’t have to depend on others. It is not nice to have to depend on others. I don’t like that very much, you have to arrange your everyday life and your behavior… I find that very annoying. **Male patient, 75 years***

*Feeling lost in the sector transition* is based on participants and the relative describing information as hard to find and a lack of coordination in the healthcare system upon discharge. All participants called for information that was tangible, easy to understand, access, and could be brought home and across sectors. The HCPs also emphasized the importance of preparing and clarifying that recovering was going to be tough and fatiguing.

*… to have a complete package when they are discharged with information that they can take out again, and which relatives also can read … **Female hospital PT, 28 years*** **.**

*I was alone, I come home all alone, it was too far out there, I could have used 2 more days (ref. at the hospital) … the next day, I should have been there for an MRI scan, but I didn’t know because I hadn’t been on the computer, there was no message, so it was too late when I discovered it, and that's not good enough. **Male patient, 74 years***

*Everything we know about stroke and the subsequent process, we might as well pass on to them at discharge. We don’t get to give them the knowledge that there will be fatigue, that there will be a psychological reaction. **Female hospital nurse, 43 years***

*Early initiation of process and tailored rehabilitation* emanates from perspectives that PA had to be initiated early, tailored to the individuals, their specific needs and preferences to be most effective and motivating. The HCPs agreed that rehabilitation had to focus on the process of recovering and returning to their former lives as rehabilitation was not a singular stage.

*(ref. to PA) Get it started quite quickly before one get lulled into something else called “I can’t because I’m sick”. **Female municipal PT, 41***

*(ref. to PA) You really have to adapt it, so that they get a good experience, it may be the way you catch them in “this is the way forward, it is well done” but it requires something. **Male hospital OT, 41 years***

The final theme *Environment as support and motive power* originates from participants describing the physical environment as affecting the desire to be physically active at the hospital or at home. Moreover, participants stated that other stroke survivors with T2DM, relatives, and HCPs played a central role in providing motivation and emotional support to take care of their health and be less sedentary.

*I couldn’t carry the water jug inside. Then I had to call my neighbor, so he came and placed water on the terrace so I could water all my plants. **Male patient, 74 years***

*In any case, they cannot get the idea themselves, they have to have support and get it (ref. to PA) incorporated into their everyday life. **Female municipal OT, 48 years***

### Building the intervention

Based on (1) a narrative review of relevant literature; (2) findings from the present study; (3) consultation with experienced clinicians and within the researcher team the “*Everyday Life is Rehabilitation*” (ELiR) intervention was developed. The ELiR intervention is a tailored 12-week home-based behavior change intervention delivered on a double-page paper instrument containing (1) action planning and goal setting, (2) motivational interviewing, (3) education on SB, PA, and sector translation, and (4) fatigue management. The instrument will work as a conversational, inspirational, and goal-setting instrument tailored by participants filling in their answers. The intervention consists of two consultations 3–5 days and 6 weeks after discharge between the participant and an HCP in their home. The participant will have the instrument handed out with additional information upon discharge from the hospital allowing them to read the instrument before the first consultation. The intervention was designed to function in hospital or during rehabilitation as an urgent need for a cross-sectoral instrument became apparent through the focus group interviews (Figure 3).

**Figure 2:**
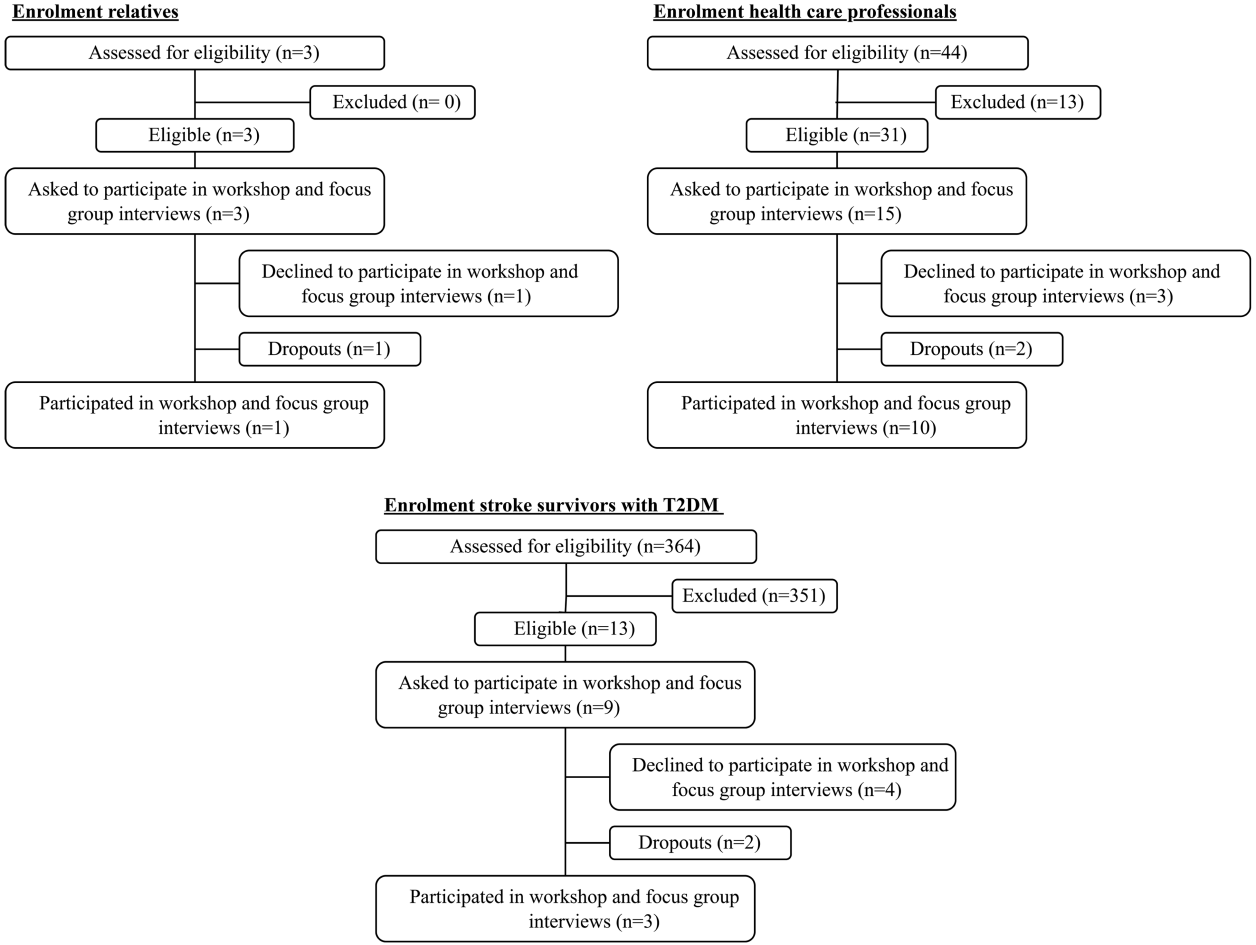
Enrolment flowchart.

**Figure 3:**
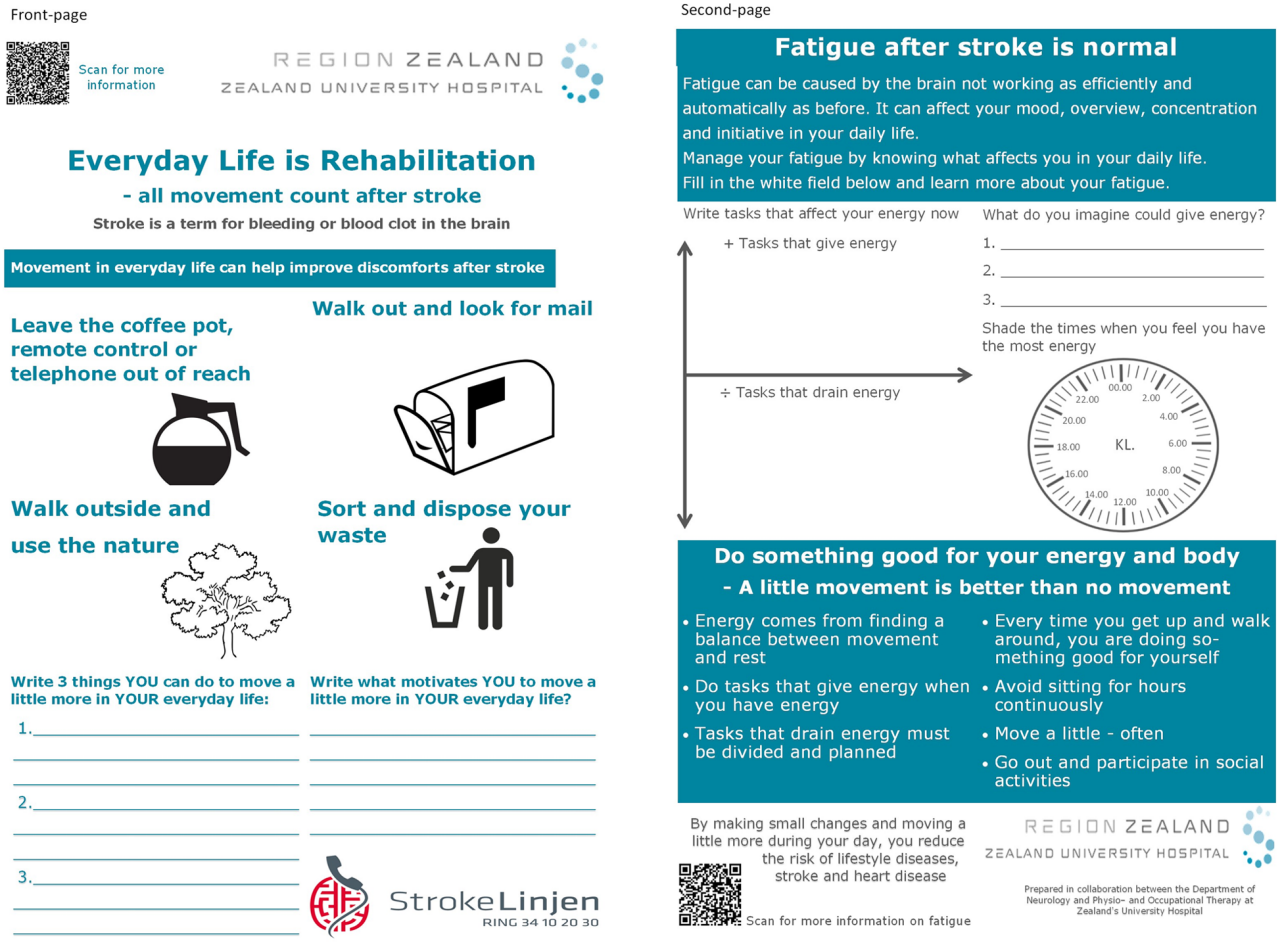
The “Everyday life is Rehabilitation” instrument.

*Action planning and goal setting* were incorporated on the front-page (Figure 3) emerging from the themes “*Everyday life is rehabilitation” and “Early initiation of process and tailored rehabilitation”* as the participants unanimously stated the need for individualization of rehabilitation to their everyday life and allowing them to decide which ADL will be modifiable to ensure sustainable changes. It was important for the participants to have the actions and goals written for them to be committed to and for HCP to follow up on. Action planning and goal setting were found during the narrative review to be effective in interventions for reducing SB and increasing PA (21, 22, 26, 27) which inspired the use in ELiR. For execution, the front-page (Figure 3) has pictograms with examples of ADL, which could be altered and thereby facilitate more movement, and a section to note three ADL movement actions and goals meaningful to each participant.

*Motivational interviewing* techniques (53) will be used to identify the participant's current SB and PA behavior and the interviewer will help the participant to understand how their behavior affects their health using the instructions (Supplementary file S3). Further, the interviewer focuses on helping the participant describe their motivation for changing their behavior and helping them note their motivation for more movement e.g., staying independent or being able to play with their grandkids. The motivational interviewing was incorporated into the ELiR based on the themes “*To preserve oneself”* and “*Environment as support and motive power*” and as it is feasible (22, 26) and effective (54)*.* This was implemented on the front-page (Figure 3) as the participants agreed it was important to identify motivational factors.

*Education on SB, PA, and sector transition* was needed as the participants described information as hard to find and described feeling lost in the sector transition emerging in the theme “*Feeling lost in the sector transition”.* The front-page (Figure 3) contains a QR code linking to information on stroke and sector transition. The second-page (Figure 3) has bullet points on how movement positively affects health and fatigue along with a QR code linking to an educational video on fatigue.

*Fatigue management* was included as all participants agreed it was important to handle fatigue as it affects all parts of one's life and behavior and is a major barrier to PA and behavior change as presented in the third quote. However, no high evidence-based fatigue management tool was found (55). In an attempt to map fatigue tendencies, the second-page (Figure 3) contains 1) a diagram to note ADL that give and drain energy, 2) noting activities that would optimize energy and 3) noting on a clock face time points throughout the day where the participant feels most energized. These elements were implemented into the ELiR based on experiences from an OT with more than 20 years of experience in fatigue management and literature (55–58), which describe written tasks and activity management as effective tools. This may help participants in clarifying what affects them during their everyday along with giving the HPCs an insight into how and when to help them manage their fatigue and behavior change.

The ELiR intervention has an appertaining instruction with a concrete guide to standardize the intervention (Supplementary file S3). All aspects of the ELiR intervention were discussed within the researcher team, face-validated with other stroke survivors with T2DM, the same HCPs from the hospital and municipal rehabilitation and an OT with more than 15 years of fatigue management experience.

## Discussion

The cross-sectoral ELiR instrument is based on the five identified themes in this study where stroke survivors with T2DM described wanting to do what they used to in order to preserve oneself, that movement should be integrated into ADL for the everyday to be rehabilitation, wanting information and support during the sector transition along with that fatigue should be identified and managed as it was a barrier to movement. By using one instrument and having two consultations, the intervention is relatively minimalistic potentially making it easy to implement and use in a hospital, rehabilitation, or community setting in the future.

### Integrating movement into activities of daily living

The participants of this study did not want to change their everyday life, however, stated that reduction of SB and PA should be implemented into their everyday life which are self-contradictory. This may explain the inconsistent methods and results of studies exploring the effect of reducing SB and increasing PA using behavioral or lifestyle interventions in stroke or T2DM populations (21, 29–31). Saunders et al. (21) reported in a systematic review that multi-component lifestyle interventions, SB, and PA interventions did not reduce mortality, cerebrovascular events or sedentary time. In a systematic review by Aguiar et al. (31) some interventions in stroke populations were effective in improving daily PA when including e.g., aerobic exercise, resistance training, home-based exercise, and health information. This was likewise the case in individuals with T2DM where regular exercise and diet interventions were effective in improving fasting glucose and exercise outcomes (29, 30). The above-mentioned intervention components, which may not be realistic to implement in a municipal setting, differ from this intervention, which focuses on movement adapted to ADL rather than e.g., aerobic exercise or resistance training. In addition, the ELiR intervention focuses on total PA throughout the day, which was reported to be associated with glucose and insulin sensitivity in stroke survivors (33). However, further research on SB in stroke survivors is warranted since high-quality studies are missing, as are interventions including action planning, inclusion of the home environment, and education (21) which are elements in the ELiR intervention.

### Fatigue management to reduce sedentary behavior and increase physical activity

Previous interventions on SB and PA in individuals with T2DM and stroke survivors were all feasible and safe with elements of tailoring, goal setting, education, and counseling which are similar to the elements in the ELiR intervention. Depression and fatigue are both interdependent and prevalent in up to half of every individual with T2DM and stroke survivors (7–10, 55, 59). Fatigue was reported (34, 35) and described as a barrier to movement by the participants and low levels of PA are associated with a higher risk of post-stroke depression (9). Hence, tailoring and goal setting of the ELiR intervention is important to help the participants change behavior and implement more movement despite fatigue and by that potentially reduce risks of depression and other related health issues. To do this participants define and note which ADL can be modified to change behavior which facilitates and ensures that the intervention will be as individual as possible making it more likely to be as successful as other interventions.

Education on the harm of SB and gain of PA has earlier been used for helping the participant to understand how their lifestyle affects their health (22, 60). The findings in this study suggest that the participants were fully aware of the harmful effects of their lifestyle. However, participants call for a different approach in tailoring rehabilitation efforts as they described themselves as too fatigued to act and that interventions often not seemed to be incorporable into everyday life. Fatigue was also reported to prevent breaking up prolonged SB and as a barrier to rehabilitation adherence (35, 37, 38, 61, 62). However, fatigue management has not yet been incorporated effectively into intervention studies in stroke survivors (63) and clinicians depend on their own experiences (57) even though post-stroke fatigue may be aggravated by SB and helped by PA (18). Therefore, the ELiR intervention focus on the positive effects of breaking up prolonged SB and on encouraging the participants to be aware that every move counts when incorporating PA despite fatigue. This approach is likewise recommended for adults with chronic conditions, which may be more doable than structured PA (20).

### An operational tool

Previously reported studies (21–23, 26, 27, 64) mentioned above have explored more comprehensive interventions compared to the ELiR intervention with regard to equipment and interactions between participants and HCP with limited success. Most interventions used more than two consultations on behavior change strategies, goal setting, education, and supervised training (21–23, 26, 27, 64) in contrast to the ELiR intervention which contains two consultations. This may influence the efficacy of the intervention due to fewer interactions, more dropouts, and participants having to take responsibility for their own health. However, it may also make the intervention more manageable for HCPs, implementable in the clinic, and more achievable for the participants as adherence to PA and home exercise programs are low (61, 62).

The minimalistic scope of the ELiR intervention was prioritized as the HCP stated to need something tangible, user-friendly, and easy to implement into their practice that was not time-consuming and expensive, thus making the rehabilitation centers in municipalities more likely to implement the intervention as a standard approach

### Limitations, strengths, and future directions

Different approaches for co-creating interventions have been utilized (25–27), with strengths and limitations to every approach, yet it is important to adapt the comprehensiveness of the co-creation process to the setting of the study (65). The trustworthiness of the findings in this study was enhanced by using a well-described framework, an interview guide similar to previous studies (36, 37) along with triangulation using multiple qualitative methods for data collection (observations, field notes, and interviews). Further, researchers analyzed the transcribed interviews separately and subsequently synthesized and identified similarities and differences (52).

Of the screened patients admitted to NC, thirteen were eligible which was relatively few due to in-hospital rehabilitation being performed at other hospitals. Only male stroke survivors and one female relative were included which were not necessarily representative of the target population and may not be adequate for reaching data saturation. However, data saturation was not viewed as a necessity for continuing the study as stroke survivors were hard to recruit and the intention was to gain insight into everyday life post-stroke. This small and homogenous representation may create gender-bias and affect the co-creation process by them being underrepresented compared to HCPs possibly causing the stroke survivors statements to be supplementary rather than co-creative. Nevertheless, the three stroke survivors' statements were, in terms of content for the development process, prioritized higher than HCP's statements to compensate for the participant ratio. Thus, the HCP's statements substantiated and supported the statements of the stroke survivors and placed those into a clinical perspective. In future studies it is important to plan the recruitment of participants for the co-creations process, in order to secure the planned representation of e.g., stroke survivors. The workshop and focus group interviews took place two to four weeks after discharge which is within the intervention period enhancing the relevance of perspectives. Participants may represent a resourceful part of the population as co-creation demands high levels of attendance and participation (66). The representation of HCPs was extensive and adequate with attendance from multiple sectors and professions.

The intention is that the ELiR intervention should be individualized based on participants' resources and easy to follow-up and bring to rehabilitation sessions. Thereby, the instrument was co-created to provide a tailored instrument to enhance the efficacy of the rehabilitation and improve communication across sectors. These findings may be transferable to other chronic patients in a similar context even though the findings also represent data exclusive to this patient group. The feasibility and efficacy of the intervention will be tested in subsequent studies to further inform the development and usability.

## Conclusion

A theoretical, co-creation framework was systematically used in this study to develop a tailored 12-week home-based behavior change intervention. The process included stroke survivors with T2DM, relative, and HCP's perspectives, and targets the implementation of movement into activities of daily living along with fatigue management in reducing sedentary behavior and increasing physical activity. Stroke survivors with T2DM, relative, and HCPs were actively engaged throughout the co-creation process increasing the likelihood of an acceptable and implementable intervention.

## Data Availability

The raw data supporting the conclusions of this article will be made available by the authors, without undue reservation.
