# Supplementary material for: Development of a tailored intervention targeting sedentary behavior and physical activity in people with stroke and diabetes: A qualitative study using a co-creation framework
Source: Front Rehabil Sci. 2023 Feb 13;4:1114537. doi: 10.3389/fresc.2023.1114537 (PMC9968882; doi:10.3389/fresc.2023.1114537)
Supplement: Supplementary file 1 [file Table1.docx]

Table S1 - Interview guide for stroke survivors with type 2 diabetes, relatives, and health care professionals

| **Questions for stroke survivors with T2DM and relatives** |
| --- |
| - Please, try to describe what a typical day looks like for you. |
| - When you hear sedentary behavior, what does it mean to you? |
| - What motivates you to take care of your health and be more physically active? |
| - What is important to you in terms of recovering from stroke and increasing your daily activity level? |
| - How do you consider physical activity or rehabilitation? |
| - How can others support you to sit less and be more physically active? |
| **Questions for health care professionals** |
| - What should be the focus when working with stroke survivors with T2DM? |
| - How do you support stroke survivors with T2DM to take care of their health and life after stroke, especially in the sector transition? |
| - Based on your experience, what motivates stroke survivors with T2DM to be more physically active at home? |
| - How do you make the efforts of the health care system more tailored? |
| - How can we influence the environment to facilitate movement during admission or at home? |
| - How do we identify what is most meaningful to stroke survivors with T2DM in their everyday life? |
