# Supplementary material for: Development of a tailored intervention targeting sedentary behavior and physical activity in people with stroke and diabetes: A qualitative study using a co-creation framework
Source: Front Rehabil Sci. 2023 Feb 13;4:1114537. doi: 10.3389/fresc.2023.1114537 (PMC9968882; doi:10.3389/fresc.2023.1114537)
Supplement: Supplementary file 2 [file Table2.docx]

| Meaning units | Categories | Themes |
| --- | --- | --- |
| If I think you have to do this and that instead of what you normally do, then it's like I'm imposing a job that one does not want […] It has nothing to do with that I have to look like a 20-year-old with big muscles. I just have to move and I will continue to do so for as long as I can.  **Male patient, 82 years**  Patients should do nothing more, very often, they are overwhelmed when they come home. They have been hospitalized and didn’t have to cook, wash clothes or walk the dog […] The rehabilitation has to be a part of what they have to do anyway because otherwise it's another thing on top and they cannot handle it. **Female municipal OT, 48 years** | Preservation of self-determination  To keep moving  Overwhelmed upon coming home  Rehabilitation as part of what they already have to do | Everyday life is rehabilitation |
| (ref. to what is meaningful to him) […] to get out of bed, and then simply just live life […] Because of the stroke, I have been very tired, so I have slept a lot, you get tired from it, really tired. **Male patient, 74 years** | Maintain independence  Tired from stroke | To preserve oneself |
| I have a hard time getting my daily routine, I usually think very clearly and logically, I do not do that right now and the things I have to do, go on the computer take forever, and sometimes I simply have to give up […] When you have a stroke, you are extremely tired afterward and it is almost impossible to make two visits to the outpatient clinic, I can hardly handle it. **Male patient, 75 years**  […] say to patients; when you come home you will cry, you will be sad, and have to talk to your loved ones. If we could just warn them and it could be part of the medicine dispensing. **Female hospital nurse, 43 years** | Giving up on keeping track of appointments  Stroke affects the energy surplus  A hard transfer from hospital to home | Feeling lost in the sector transition |
| It is a process to make coffee. If you spill or if it tastes bad then you have to try again. But you have not lost anything by it, you have been training, you have tried, you have kept your balance, you have used your arm, you have been able to pour, so you can try again, it is a process. **Male hospital OT, 41 years** | It is a process  Try again if you are unsuccessful | Early initiation of process and tailored rehabilitation |
| We have family support, someone who helps and says you can do it. I believe in that. I think the lonely people, who sit alone at home […] and do not have anyone who is there for them, then I think it is even harder to recover. **Female relative, 76 years**  Patients can mirror each other, they can find motivation, they can create some unity and they can get a sense of “well he can do that, then maybe I can too” or we can talk things through. **Female municipal PT, 41 years** | Relatives as a resource  Finding motivation in someone in the same situation | Environment as support and motive power |
