# Supplementary material for: Development of a tailored intervention targeting sedentary behavior and physical activity in people with stroke and diabetes: A qualitative study using a co-creation framework
Source: Front Rehabil Sci. 2023 Feb 13;4:1114537. doi: 10.3389/fresc.2023.1114537 (PMC9968882; doi:10.3389/fresc.2023.1114537)
Supplement: Supplementary file 3 [file Table3.docx]

**Instructions for "Everyday Life is Rehabilitation"**

"Everyday Life is Rehabilitation" is developed on perspectives of patients', relatives', and health care professions and designed to increase quality of life and health through movement in individuals with diabetes who have survived a stroke. "Everyday Life is Rehabilitation" can be used in the context of rehabilitation and prevention.

It is the intention that individuals with diabetes who have survived a stroke can read, understand and use "Everyday Life is Rehabilitation" without any health education. In addition, "Everyday Life is Rehabilitation" should provide inspiration for movement in everyday life and fatigue management after a stroke.

Health care professionals can use "Everyday Life is Rehabilitation" as a conversation tool or inspiration for conversations associated with rehabilitation and prevention, focusing on reducing sedentary behavior and increasing light physical activity when the individual is discharged to their home.

**Instructions for health care professionals:**

If "Everyday Life is Rehabilitation" is filled out, the written are discussed with the individual.

If "Everyday Life is Rehabilitation" is *not* filled out, the following instructions should be used:

(The individual should complete **4, 5 and 6** at the earliest 3 days after discharge from the hospital)

1. Describe the purpose of the conversation about "Everyday Life is Rehabilitation" to the individual.

Say: "*The purpose of this conversation is to determine how you can move more in your everyday life and thereby help your discomforts, including possible fatigue after your stroke"*

1. Ask the individual to read the front-page, look at the pictograms and write 3 things they can do in their everyday life to move more.

Question: *"With inspiration from the pictograms, when you think of your everyday life, what everyday activities could be changed* *so that you move more in your everyday life?"*

1. Ask the individual to write down what motivates them to move more in their everyday life.

Ask: *"What motivates you to move more in your everyday life?"*

1. Ask the individual to read the entire second-page about fatigue and fill out the chart.

Ask: *"After you have read the second-page, when you think of your everyday life, which of your current daily activities gives you energy or drains your energy?"*

1. Ask the individual to write down the things that give energy or that have previously energized them.

Ask: *"In your everyday life, what do you like to do that gives you energy, or what have you previously liked to do that gave you energy?”*

1. Ask the individual to mark on the clock face when, on a normal day, they feel most energized.

*Say: "Try to shade on the clock, the times you feel energized when thinking of a normal day."*
